# Supplementary material for: An integrated optimization model and metaheuristics for assortment planning, shelf space allocation, and inventory management of perishable products: A real application
Source: PLoS One. 2022 Mar 8;17(3):e0264186. doi: 10.1371/journal.pone.0264186 (PMC8903274; doi:10.1371/journal.pone.0264186)
Supplement: Attachment — Submitted filename: Review Report_PONE-D-21-28756.pdf [file pone.0264186.s002.pdf]

Review report

This study investigated an integrated optimization problem considering the assortment planning, shelf space allocation, and inventory management and supplier selection for the perishable products. A mixed integer non-linear programming model was established to formulate the NP-hard problem. An evolutionary genetic algorithm and vibration damping optimization algorithm were employed to solve the proposed model. Numerical experiments and a real-life case study were exemplified to validate the proposed model and solution methods. Overall, the authors have spent a lot of efforts on this study. However, the current version of this paper has a number of limitations that preclude its publication. In what follows we are identifying the major shortcomings of the paper:

1. There are several errors or unclear expressions in the model formulation.

1) The authors should use a unified expression (e.g., expressed by using Mathtype software or formula editor embedded in Microsoft Office) to type the equations. The inconsistence appeared in both the model formulation section and the case study section. The authors should revise the corresponding parts.

2) Some typos are not defined, such as *I*, *J*, *G*, x_{it}, s_k, V_{it}^G … Do *I/J/G* represent a number or set?

3) The author defined many types of cost; however, the difference of these cost was not clearly illustrated. For example, what is the selecting cost and what's the difference between it and the fixed cost of ordering from suppliers? What's the difference of the penalty cost of substitution for product *i* and the unit cost of non-responding to the demand of product *i*? The authors should give more detailed explanation for these costs to show readers with concrete understanding.

4) Some definitions are difficult to understand. For example, m_{ikt}^g is defined as the amount of product *i* with lifetime *g* allocated to the demand of product *k* at time *t*. Does it represent the amount of product *i* with lifetime *g* that substituted by the product *k* at time t? If yes, only this sales income was calculated? How about the sales income of directly selling the product *i*? The β_i expressed the spatial elasticity of product *i*. But there is only β appeared in equation (17). A lot of these inconsistences appears in the modeling part. The authors should proofread the whole mathematical formulation to ensure right and consistent expressions, meanwhile, give more detailed explanations or a specific example to illustrate some abstract equations, such as β_i.

5) Why is there a 2 in the denominator for the calculation of *TCI* in equation (5)?

6) It is better to point out the specific set that the variable belongs to when conducting the summation to show a clearer expression. For example, $TCO = \sum_{j\in J}\sum_{t\epsilon T} oc_j \cdot o_{jt}$ instead of $TCO = \sum_j \sum_t oc_j \cdot o_{jt}$.

2. There is no need to show all details for the encoding and decoding procedure. Only an example for the same or similar process is enough because several parts are similar. Additionally, the figure is not clear with blurry expression.

3. All the parameter settings and the information of the computer or workstation used for calculation should be fully illustrated in an appropriate place.

4. Some results and analysis may be wrong. For example, in Table 9, the value of *TP* illustrated in the second row that calculated by GA and GAMS is not consistent with the value that directly calculated by equation (1). Similar problems are appeared in Tables 6 and 7. The illustration of the gaps for GA and VDO under figure 10 is 0.6726% and 0.5974% instead of 0.6726 and 0.5974. The authors should take care of these problems and ensure accurate and authentic results.

5. What is the significance and specification of considering the perishable products instead of other products on model formulation or to the practical implication. Is the consideration of lifetime *g* for perishable products? If yes, a sensitive analysis for the lifetime should also be conducted.
