# Supplementary material for: An integrated optimization model and metaheuristics for assortment planning, shelf space allocation, and inventory management of perishable products: A real application
Source: PLoS One. 2022 Mar 8;17(3):e0264186. doi: 10.1371/journal.pone.0264186 (PMC8903274; doi:10.1371/journal.pone.0264186)
Supplement: Attachment — Submitted filename: Response to Reviewers.docx [file pone.0264186.s004.docx]

**Response to Reviewers' Comments on [PONE-D-21-28756R1] - [EMID: 6471bfd9f0373dab]**

***An integrated optimization model and metaheuristics for assortment planning, shelf space allocation, and inventory management of perishable products: A real application***

We appreciate that the reviewer’s comments. The followings are our point-by-point responses:

Reviewer #1: The authors have already addressed some of my concerns. However, for the question 4 about the inconsistence of the result in Tables 6, 7 and 9 still makes me confused. Taking Table 9 as an example, according to the equation (1), TP=TR-TCO-TCSS-TCP-TCI-TCS-TCU-TCLS. However, if we directly calculate equation with the results listed in Table 9, for example as the GA column, 184267.47-39-18-7856.75-246.46-3216.75-426.64-1625.01=170838.86 instead of the result 100538.86 listed in Table 9. In addition, as the authors have changed the equation (2), the result should be accordingly updated.

Thanks

1) Many thanks for bringing this issue into our attention. Yes, that’s correct. We check all data and table numbers and should say with a great apology, we have Typo mistakes in table numbers, and some digit has missed in typing.

We checked entire numeric results in all tables and found some mistakes. We revised these mistakes as follows:

1274.3 instead of 127.3 in table 6 (digit 4 was missed),

7769.028 instead of 769.028 in table 7(digit 7 was missed),

78156.75 instead of 7856.75 in table 9 (digit 1 was missed)

2) Thank you very much for your thoughtful comment. After this comment, we have made an attempt to update results. To this end, we run the model and found that the results are same. After a detailed review, we found that two equations are working in the same manner.

In first version, the equation 2 was$TR=\sum_{i} \sum_{k} \sum_{g} \sum_{t} p_{i}.{(m}_{ikt}^{g})$, in which $m_{ikt}^{G}$ Represents the amount of product i with lifetime g that is substituted by the product k at time t. If i=k, this case represents direct sale of product i for demand of product i.

In second version (Revision 1), to clear up the ambiguity that occurred to the referee we clarify this equation to

$$TR=\sum_{i} \sum_{k\neq i} \sum_{g} \sum_{t} p_{i}.{(m}_{ikt}^{g}+x_{i}^{t})$$

While i≠k. Consequently, it should be noticed that the results of these equations are the same and in case i=k, ${\sum_{k} \sum_{g} m_{ikt}^{g}=x}_{i}^{t}$.

A summary of comparison is given in the table below

|  | 1 | 2 |
| --- | --- | --- |
| Eq. | $TR=\sum_{i} \sum_{k} \sum_{g} \sum_{t} p_{i}.{(m}_{ikt}^{g})$ | $TR=\sum_{i} \sum_{k\neq i} \sum_{g} \sum_{t} p_{i}.{(m}_{ikt}^{g}+x_{i}^{t})$ |
| substitution matrix | $w_{ik}=\begin{matrix} 1 & w_{12} & w_{13} \\ w_{21} & 1 & w_{23} \\ w_{31} & w_{32} & 1 \end{matrix}$ | $w_{ik}=\begin{matrix} 0 & w_{12} & w_{13} \\ w_{21} & 0 & w_{23} \\ w_{31} & w_{32} & 0 \end{matrix}$ |

In result, the first version is considered in this revised paper which are able to consider direct sale.
